# Masculinity and Lying

**DOI:** 10.3389/fpsyg.2021.684226

**Published:** 2021-07-30

**Authors:** Marc Vorsatz, Santiago Sanchez-Pages, Enrique Turiegano

**Affiliations:** ^1^Department of Economic Analysis, National University of Distance Education (UNED), Madrid, Spain; ^2^Department of Political Economy, King's College London, London, United Kingdom; ^3^Department of Biology, Autonomous University of Madrid, Madrid, Spain

**Keywords:** lying, deception, cheap-talk, masculinity, testosterone, C72, C91, D83, D87

## Abstract

Dishonesty in communication has important economic implications. The standing literature has shown that lying is less pervasive than predicted by standard economic theory. We explore whether biology can help to explain this behavior. In a sample of men, we study whether masculine traits are related to (dis)honesty in a sender-receiver game. We study three masculine physical traits: the second-to-fourth digit ratio, facial morphometric masculinity and the facial width-to-height ratio. These biomarkers display significant associations with lying and deception in the game. We also explore the extent to which these effects operate through social preferences or through beliefs about the behavior of receivers.

## 1. Introduction

Truthful communication is a pillar of human interactions. Economic exchanges rely on language being trustworthy. Many buyers consult financial advisors before acquiring stocks or probe sellers on the quality of their goods. Honest communication is also crucial in policy making. Central banks make pronouncements which influence the actions of investors and stock traders. Regulatory bodies consult private entities before setting new standards. A third area where communication is crucial is organizations. Division managers, for example, report local market conditions to the their superiors who then use this information to devise their plans for the firm.

But better-informed agents often have incentives to misrepresent what they know in order to alter the decision-making process in their favor. Central bankers have an incentive to manipulate economic expectations (Stein, 1989). Private firms hired by financial agencies may recommend the adoption of standards to their own advantage (Melumad and Shibano, 1994). Low-level managers may bias their reports to maximize the profits of their division rather than of the entire firm (Dessein, 2002). Given that dishonesty in communication severely undermines trust (e.g., Gawn and Innes, 2018), it is of great importance to study its prevalence and determinants.

The experimental literature on strategic information transmission has shown that individuals engage in truthful communication above standard game-theoretical predictions (e.g., Gneezy, 2005; Cai and Wang, 2006). This literature also highlights that purely monetary cost-benefit calculations cannot explain such behavior. A substantial proportion of individuals refuse to tell lies that may benefit them at the expense of others (e.g., Sanchez-Pages and Vorsatz, 2007; Hurkens and Kartik, 2009), even if these lies can lead to Pareto-superior allocations (Erat and Gneezy, 2012)^1^.

In this paper, we offer an exploratory study of the role biological factors play in explaining the individual heterogeneity observed in honesty in strategic communication. In particular, we focus on masculine physical traits. The development of masculine physiology-related traits during key life stages is associated to organizational changes in the neural circuitry of the brain which can in turn affect behavior (e.g., Sisk and Zehr, 2005).

The study of masculine traits is particularly relevant in the context of strategic information transmission because of two reasons. First, men are typically overrepresented in environments such as firms, finance and policy making where communication of this sort is pervasive^2^. Second, a growing body of literature has shown that biological mechanisms, and sexual hormones in particular, influence moral decision-making (e.g., Capraro, 2018). As strategic communication often entails the choice between truth-telling (an almost universal moral principle) and self-serving lies (widely deemed as antisocial), masculine traits are likely to relate to this choice.

The experimental literature has shown that individuals with more masculine facial features are less trustworthy (Stirrat and Perrett, 2010) and more likely to cheat in non-strategic settings (Haselhuhn and Wong, 2012; Geniole et al., 2015). Jia et al. (2014) find that CEOs with more masculine facial features are more likely to be subject to external audits and to be accused of financial wrongdoings. But to the best of our knowledge, the present paper is the first to explore whether markers of masculinity correlate with lying and deception in strategic communication.

We conduct a laboratory experiment based on the sender-receiver game in Sanchez-Pages and Vorsatz (2007) with a sample of 168 males. Participants are matched in pairs; one is assigned to be the sender, the other to be the receiver. Only the sender is informed about the state of the world which determines players' payoffs conditional on the action the receiver will take later on. The sender sends a non-verifiable message to the receiver about the state of the world. The receiver then decides which action to take and payoffs are realized. Preferences are opposed: the best outcome for the sender is the worst for the receiver and viceversa. As a result, the standard game-theoretical prediction is that senders' messages are entirely uninformative.

In our analysis, we make use of the distinction between lying and deception introduced by Sobel (2020). Lying refers to the content of messages. Deception entails misleading others to obtain an advantage, which in our design can be achieved by lying when one expects to be trusted but also by telling the truth when one expects to be distrusted (Sutter, 2009).

We study how lying and deception by senders in this game correlate with a set of masculine physical traits. Two of the biomarkers we consider are related to testosterone exposure at two developmental periods, *in utero* (second-to-fourth digit ratio) and during puberty (facial morphometric masculinity). The third one has been associated to antisocial and dominance behavior (facial Width-to-Height ratio). We discuss these markers in detail and the debates about their relevance in the following section.

Our results suggest the existence of a significant relationship between the masculine physical traits we study and (dis)honesty in strategic communication. We find that individuals exposed to higher levels of testosterone *in utero* and with more masculine facial features are more likely to send truthful messages. However, we also find that the latter engage more often in deception through truth-telling. In contrast, individuals exposed to higher prenatal levels of testosterone seem to display a stronger lie aversion as they are more likely to engage in costly truth-telling, i.e., send a truthful message when it is expected to be trusted.

Finally, we explore whether these associations between lying and the masculine physical features we consider operate mostly through social preferences (e.g., lying aversion) or through beliefs about the behavior of receivers. Results suggest that preferences are the main drivers of these effects.

The present paper contributes to the rapidly expanding literature on the influence of biometric traits and sexual hormones on economic behavior. Studies in this area have shown that differences in circulating and basal levels of sexual hormones influence risk preferences (e.g., Garbarino et al., 2011), social preferences (Buser, 2012a; Sanchez-Pages and Turiegano, 2013), bidding in auctions (Chen et al., 2013; Pearson and Schipper, 2013; Sanchez-Pages et al., 2014; Schipper, 2015), cooperation in social dilemmas (Sanchez-Pages and Turiegano, 2010; Cecchi and Duchoslav, 2018) and willingness to compete (Buser, 2012b; Wozniak and Harbaugh, 2014). The closest papers to ours in this strand of the literature have studied the effect of administered testosterone on non-strategic misreporting (Wibral et al., 2012) and on strategic gambling in poker (van Honk et al., 2015). In contrast to these two papers, we consider stable physiology-related traits rather than hormone infusions.

There are two other papers related to ours which explore the correlation between biological data and honesty in sender-receiver games. Using eye-tracking techniques, Wang et al. (2010) observed that senders look disproportionately at the payoffs corresponding to the true state of the world and that their pupils dilate when they send deceptive messages. On the other hand, Volz et al. (2015) studied the neural correlates of dishonesty using fMRI and found that brain activation patterns can reveal whether the sender intends to deceive the receiver.

Finally, our paper relates to the literature on gender differences in lying in sender-receiver games. If masculinity and femininity are viewed as a continuum, we would expect our results to reflect to some extent any gender differences observed in these studies. A recent meta analysis on deception games^3^ by Capraro (2018) showed that male senders are more likely to lie than female senders when lies benefit them at the expense of the receiver and when lies hurt the sender but benefit the receiver. We obtain results along these lines when studying deception and costly truth-telling, in the sense that individuals exposed to more prenatal testosterone and with more masculine facial features engage more often in these behaviors. However, for the purpose of our study, gender is a too coarse marker of physiological differences as it is binary and it is heavily influenced by socialization.

## 2. Masculine Physical Traits

Masculinity can be defined as "a set of physical and behavioral traits that are male typical" (Lippa, 2016). These traits can distinguish men from women and/or order men by their degree of male typicality. Masculinity is thus not a latent trait but a set of dimensions that are typical of men. From all possible masculine traits proposed in the literature, the ones we choose in our study are based on rather stable physical features.

A widely studied physiological masculine trait is exposure to androgens -testosterone in particular- during key phases of development. Androgens produce distinctive changes in the male body, such as greater musculoskeletal development and the appearance of secondary sexual characteristics. More importantly, they have organizational effects on the brain, that is, they modify neural structures and can therefore influence adult behavior (Sisk and Zehr, 2005). In particular, testosterone seems to affect the structure of the amygdala, a cluster of neurons responsible for emotional reactions such as responses to interpersonal challenges and threats (van Honk et al., 2012).

There are two stages of development during which androgen exposure has crucial organizational effects on the brain: the prenatal period and puberty (Schulz et al., 2009; Berenbaum and Beltz, 2011). Androgens levels during these two periods have been proxied in the literature with two types of morphological features, the second-to-fourth digit ratio (2D:4D) and facial morphometric masculinity (fMM).

### 2.1. 2D:4D

The second-to-fourth digit ratio (2D:4D) is the ratio between the length of the index and the ring fingers. The available evidence suggests that the 2D:4D ratio is related to the ratio of amniotic testosterone/estrogen concentrations (Zheng and Cohn, 2011; Swift-Gallant et al., 2020). A lower 2D:4D ratio indicates higher relative exposure to masculine sexual hormones during foetal development. Men across countries have shorter ratios than women (Hönekopp et al., 2007; Grimbos et al., 2010). These differences are already present in human embryos (Galis et al., 2009). The underlying mechanism seems to be that both digit growth and the development of primary sexual characteristics are influenced by the Hox genes (Manning et al., 1998). Although early studies showed a correlation between 2D:4D and circulating (current) testosterone in adults, more recent ones have conclusively rejected that association (e.g., Honekopp and Watson, 2010).

Several studies have cast doubts on the validity of 2D:4D as a proxy for prenatal testosterone exposure. These studies find no correlation between 2D:4D and testosterone levels in umbilical blood or mother's blood (Hickey et al., 2010; van Leeuwen et al., 2020). However, these methods to measure foetal hormonal levels are imprecise. In mammals, testosterone levels at birth measured from the umbilical cord are substantially lower than during pregnancy. In addition, the role of the placenta in the process of blood exchange with the mother is to regulate the hormone levels the foetus is exposed to. In contrast, there is abundant indirect evidence of 2D:4D correlating with prenatal testosterone coming from studies of patients with congenital adrenal hyperplasia, Klinefelter's syndrome and androgen insensitivity syndrome (for meta analyzes, see Honekopp and Watson, 2010; Richards et al., 2020; Sadr et al., 2020). This evidence plus the lack of competing explanations (Swift-Gallant et al., 2020) lead us to believe that that 2D:4D remains the best available proxy for testosterone levels during foetal development^4^.

The relationship between 2D:4D and strategic behavior is not fully understood yet. Earlier studies showed that men with lower 2D:4D are more prosocial and cooperative (Millet and Dewitte, 2006, 2009; van den Bergh and Dewitte, 2006). Later, large studies found no evidence of sexual hormones affecting decision making (e.g., Zethraeus et al., 2009; Ranehill et al., 2018) and economic preferences (Neyse et al., 2021). Recent studies suggest that the role of 2D:4D is very context-dependent (e.g., Ryckmans et al., 2015; Cecchi and Duchoslav, 2018), especially when the context challenges individual status (Millet, 2011; Manning et al., 2014; Millet and Buehler, 2018). One possible reason for this is that circulating testosterone, which seems to activate the neural circuitry affected by prenatal testosterone exposure, varies with environmental stimuli (Montoya et al., 2013). Several studies support the idea that the effects of testosterone levels are modulated by circuits established by prenatal exposure to sex hormones (van Honk et al., 2011a, 2012; Buskens et al., 2016). Evidence from brain imaging underscores that prenatal exposure to sex hormones matters for both neural and behavioral manifestations of testosterone in adult behavior (Chen et al., 2016). Meta analyzes linking this trait with violent and aggressive behaviors (Honekopp and Watson, 2011; Turanovic et al., 2017) also suggest that 2D:4D may affect economic decisions.

Following the standard procedures in the literature (e.g., Pearson and Schipper, 2012) we scanned both hands of all participants. Using the TPS morphometric software (Rohlf, 2015) on these images, two researcher assistants took digit length measures of the second and fourth digits of each hand from the flexion crease proximal to the palm to the top of the digit. Interrater correlation was *r* = 0.747 for the right hand and *r* = 0.732 for the left hand. The average of the four values is the first of our markers of interest. In our analyzes below we have transformed the variable so that higher values are meant to signify higher exposure to prenatal testosterone.

### 2.2. Facial Morphometric Masculinity

Although there are no direct measures relating pubertal hormone levels to the facial shape, it is well established that higher androgens levels during puberty are related to facial bone size and certain facial features (e.g., Marečková et al., 2011). Given that testosterone exposure in adolescence creates sex differences in the face shape, another masculine physical trait to be considered is the degree of difference between a man's face and a female face of reference.

**Figure 1:**
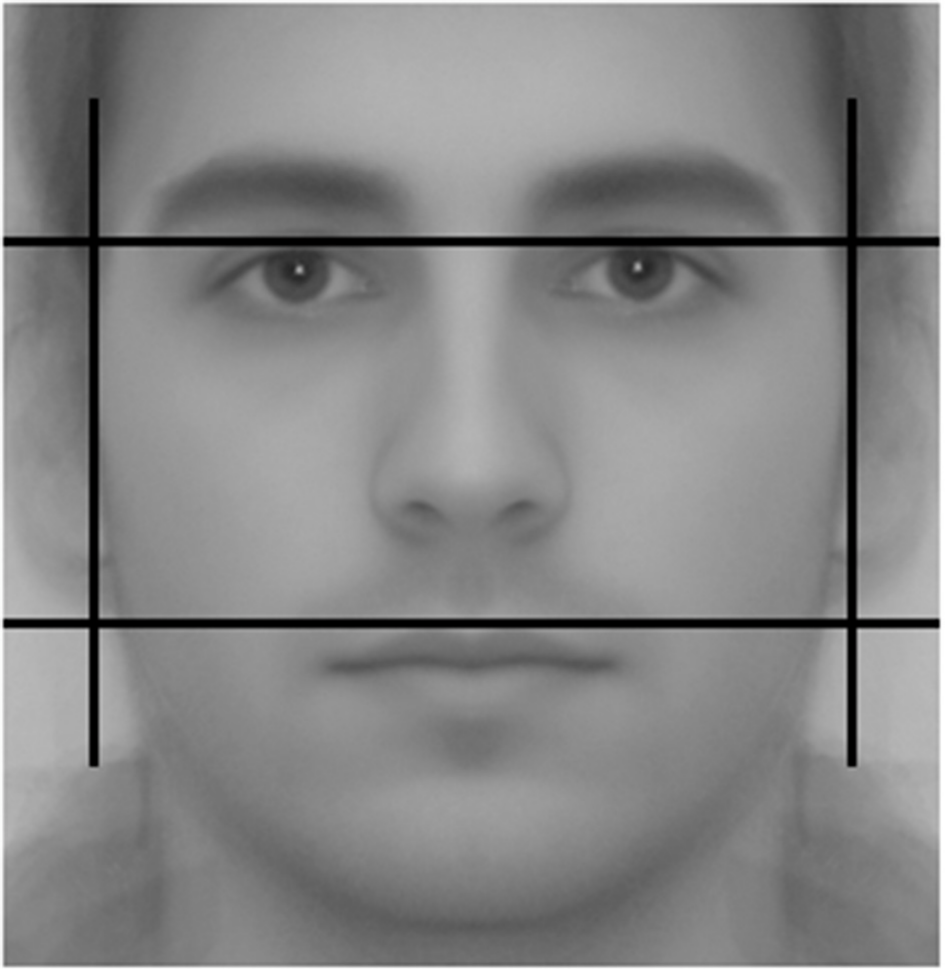
Facial measures used to compute fWHR.

There is a wide variety of methods to measure facial dimorphism^5^. Specifically, we employ facial morphometric masculinity (fMM), which is in line with others employed in the literature (van Dongen, 2014; Ekrami et al., 2021). In previous studies, we found an association of fMM with rejections of low offers in the ultimatum game (Sanchez-Pages and Turiegano, 2013), and more aggressive bidding in the first price auction (Sanchez-Pages et al., 2014). One key advantage of morphometric methods is that they gather information from the entire facial shape rather than from specific distances or angles. Specifically, fMM corresponds to the Procrustes distance between the shape of the participant's face and a reference female face. This distance is computed from a number of landmark coordinates placed on the facial image. Two research assistants independently placed 39 of these landmarks (LMs) in the image resulting from averaging the two photographs of each subject. These LMs can be unambiguously identified in every photo (see Figure 1) and are thus comparable across individuals. Since we are interested in the changes in the facial shape induced by the exposure to testosterone during puberty, LMs were not placed on soft parts of the face, which are more prone to changes during life. We built the female reference image by averaging the photos of 100 females of similar age and background to the subjects in our sample. The TPS software (Rohlf, 2015) computed a fMM score for each individual with higher scores indicating a higher distance between the subject's face and the average female face, that is, higher facial masculinity. This software also implements a correction accounting for LM placement error across researchers. The resulting score is our second trait of interest.

### 2.3. Facial Width-To-Height Ratio

The available evidence suggests that men with certain facial features tend to be more aggressive and less prosocial (Geniole et al., 2015; Haselhuhn et al., 2015). Some of these features are based on raters' perceptions whereas others are calculated from physiological markers. Perceived masculinity is problematic because subjective judgments tend to be influenced by perceived health and skin color. Objective measures are better suited for our purposes. The facial width-to-height ratio (fWHR), first described by Weston et al. (2007), is probably the most popular among these because it is very easy to compute: fWHR is the ratio between the width and the height of the face.

Individuals with higher fWHR engage more often in threat and dominance behaviors and are perceived as more threatening and dominant (Geniole et al., 2014). They also are more prone to engage in antisocial behavior (for meta analyzes see Haselhuhn et al., 2014, 2015) and display superior deception skills (Matsumoto and Hwang, 2021). Elite hockey players with higher fWHR are sanctioned with more penalty minutes over the season (Carré and McCormick, 2008). Since fWHR is also associated with dominance in non-human primates (Lefevre et al., 2014), some authors have argued that the trait serves, or at least served in our evolutionary past, as a signal of aggression and dominance in inter-male competition (Geniole et al., 2015; Wang et al., 2019). In economic games, this marker has been shown to correlate with the propensity to exploit others in the trust game (Stirrat and Perrett, 2010; Sanchez-Pages et al., 2014).

**Figure 2:**
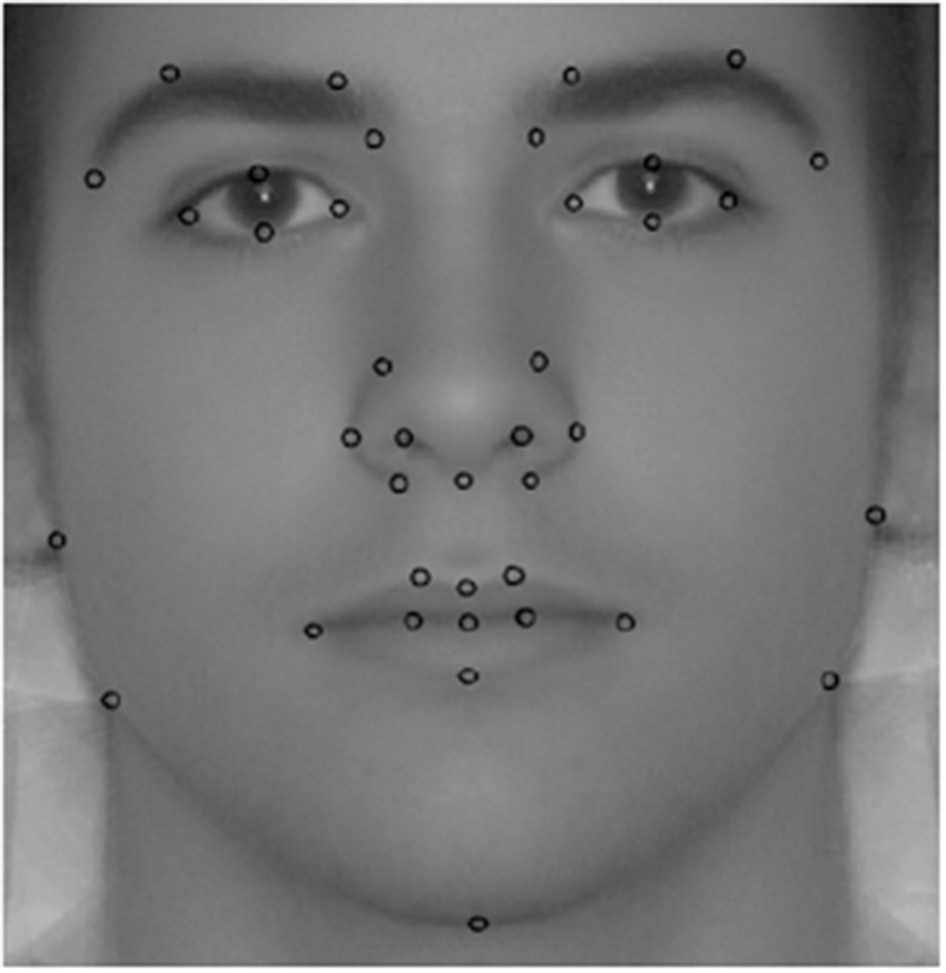
Facial landmarks used to compute fMM.

To construct the fWHR, we took two full frontal facial color photographs of our subjects at standardized light and distance conditions. They were asked to remove any facial adornments and were carefully instructed to look into the camera with a neutral expression. We later converted these images to a 8-bit gray-scale format. Using the TPS morphometric software and following the method described in Weston et al. (2007), two research assistants measured the maximum horizontal (bizygomatic) distance from the left to the right cheekbone and divided it by the vertical distance between the lip and brow (see Figure 2). The correlation between their measures was *r* = 0.840. The average of the ratios obtained by the two researchers is our third and final masculine trait of interest.

It is important to note at this point that the available evidence strongly suggests that fWHR is not sexually dimorphic (e.g., Kramer, 2017). This casts some doubts on the value of fWHR as a masculine trait. The literature suggests that this lack of sex differences might be driven by the influence of body weight on the facial shape. For that reason, we also collected height and weight measurements of our subjects to construct their Body Mass Index (BMI) and we included it as a control in all our specifications.

## 3. The Experiment

### 3.1. Design

#### 3.1.1. Equilibrium Predictions

Our experimental design is based on Sanchez-Pages and Vorsatz (2007). First, nature randomly selects one of two tables, A or B, with equal probability. The chosen table θ∈{*A, B*} determines how payoffs will be realized. There are two players, the *sender* and the *receiver*. Only the sender is informed about θ. After being informed about the table selected, the sender sends a message to the receiver telling him^6^ which table nature selected. Formally, the sender chooses a mixed strategy profile ${\left\{p\left(m|\theta \right)\right\}}_{\theta =A,B}^{m=A,B}$ where *m*∈{*A, B*} is the message sent with *p*(*A*∣θ)+*p*(*B*∣θ) = 1.

The receiver observes the message *m* and must choose a mixed strategy over his available actions, A and B. The action taken *s*∈{*A, B*} is relevant for both players as it determines in conjunction with the table selected θ the payoffs they receive. The payoff structure is of divergent interests (Crawford and Sobel, 1982) as shown in the matrices in Table 1. This means that the best action for the receiver is the one that matches the table selected, i.e., *s* = θ. The opposite holds for the sender. Lying occurs when the sender sends the message “The table selected is Table 1A (B)” when nature has actually selected Table 1B (A), i.e., when *m*≠θ.

**Table 1:** Payoff matrices.

**Action A**	**Action B**
**Table A**	
40 for the sender	100 for the sender
100 for the receiver	40 for the receiver
**Table B**	
100 for the sender	40 for the sender
40 for the receiver	100 for the receiver

The receiver holds a belief profile ${\left\{\mu \left(s|m\right)\right\}}_{s=A,B}^{m=A,B},$ where μ(*m*∣*m*) is the probability with which the receiver believes that the message *m* is truthful and action *s* = *m* will indeed earn him the highest payoff. Note that μ(*A*∣*m*)+μ(*B*∣*m*) = 1. Denote the mixed strategy of the receiver as ${\left\{q\left(s|m\right)\right\}}_{m=A,B}^{s=A,B},$ where *q*(*A*∣*m*)+*q*(*B*∣*m*) = 1. As it is customary in the literature, we will interpret that a receiver trusted (or followed) the sender's message if he took the action that maximized his payoff if the message was truthful, i.e., when *s* = *m*.

Under these preferences, the standard game-theoretical prediction is that the set of sequential equilibria of the game are all "babbling": Senders send each message with the same probability regardless of the table chosen, i.e., *p*(*A*∣*A*) = *p*(*A*∣*B*) = *p*∈[0, 1], meaning that they lie with 50% probability. This renders messages completely uninformative, so receivers' posterior beliefs remain identical to the prior, i.e., $\mu \left(A|m\right)=\mu \left(B|m\right)=\frac{1}{2}$. Given this behavior on the part of senders, receivers should follow messages with 50% probability. Note that 1) risk attitudes do not alter this set of predictions and 2) the babbling equilibrium with $p=\frac{1}{2}$ is the unique logit agent quantal response equilibrium of the game (McKelvey and Palfrey, 1995)^7^.

#### 3.1.2. A Behavioral Taxonomy

Let us now introduce some behavioral considerations. Suppose that the sender expects the receiver to trust his message with more (less) than 50% probability. In that case the sender should tell a lie (the truth) under standard preferences (and independently of his risk attitude). It is at this point where we should make a crucial distinction between lying and deception: whereas lying is related to the *content* of the message, deception relates to the *outcome* the message is trying to induce (Sobel, 2020). Obviously, lying occurs in our design when a subject sends an untruthful message, i.e., *m*≠θ. On the other hand, we will say that a sender engages in deception when he sends a message aiming to induce the receiver to take the inferior action, that is, the best action for the sender (note again that we are assuming that receivers take the action they believe maximizes their own payoff). Therefore, a sender can be deceptive in our experiment either by lying when he expects the receiver to trust his message with more than 50% chance or by telling the truth when he expects the receiver to follow his message with less than 50% chance^8^.

On the other hand, a sender who tells the truth when he expects the receiver to trust his message with more than a 50% chance is not maximizing his expected payoff. We will say that this sender is a *strong truth-teller*. A sender who tells a lie when he expects the receiver to distrust his message with more than a 50% chance is not maximizing his expected payoff either and in addition he is lying. Given that such sender is paying a monetary cost and probably a psychic (lying) cost also to make the receiver obtain a higher payoff, we refer to this sender as an *altruistic liar*^9^ Table 2 summarizes this behavioral taxonomy.

**Table 2:** Behavioral taxonomy by messages and beliefs.

**Message \ Belief**	**Trust <50%**	**Trust > 50%**
**Truthful**	Deception	Strong truth-telling
**Untruthful**	Lying	Lying
	Altruistic lying	Deception

### 3.2. Procedures

The study was conducted with undergraduate students at the Universidad Autónoma de Madrid (UAM), Spain, in early 2016. It was approved by the UAM Research Ethics Committee (reference CEI 62-1086). Subjects were recruited from the subject pool of the Madrid Laboratory for Experimental Economics (MADLEE) and with posters and flyers distributed within the Faculty of Sciences where the experimental sessions took place. The invitations and promotional materials mentioned that participants would be taken images of their faces and their hands and that these images could not be linked to any personal information. No mention to the all-male nature of the experiment was made during the recruitment process or the sessions.

A total of 168 males participated in 10 sessions composed by 12–24 subjects each. This sample size was meant to detect the associations between economic behavior and masculine physical traits identified in previous works^10^. All subjects except one identified themselves as Caucasian; we excluded that subject from our analysis as the fMM measure requires ethnic homogeneity. Another subject did not fill the belief elicitation question in one treatment. The sessions comprised two experiments run in a fixed order with a break in the middle to collect participant's morphometric data. After these measures were collected subjects were free to go if they preferred to not participate in that second component, whichwas unrelated to the one discussed here^11^. The duration of the experiment presented in this paper was 40–60 min, including the collection of physiological measurements.

Subjects were called one by one to the lab and took sit at individual tables. Instructions were then read aloud (see the Supplementary Material). During this debriefing, participants were reminded that experimenters were to take photos of their faces and scans of their hands after the session and that these images would be anonymized. Participants were invited to leave the experiment at that point if they did not consent with these images being taken; they could keep the show-up fee if they left. They were also told they were free to leave the session at any later stage.

Subjects participated in two treatments administered in a fixed order. Subjects received no feedback between them. First, they participated in a *control* treatment, where they played the sender-receiver game described above. After that, they played a *punishment* treatment, a version of the Punishment Game in Sanchez-Pages and Vorsatz (2007). That game is identical to the one in the control treatment up to the point where the receiver takes his action. Before payoffs are realized, the receiver is informed about the payoff outcome and the table selected by nature and he is given the option to accept the resulting payoff distribution or to reduce his and the sender's payoff to zero. It is easy to see that the set of sequential equilibria of this game under standard preferences is identical to the one in the control treatment as no purely payoff-maximizing receiver would reduce his own payoff.

Participants made their choices in the two roles within each treatment. When choosing as receivers, subjects observed a message from the sender and chose their action. When choosing as senders, they where informed about the table selected by nature and decided which message to send to the receiver. We used a simplified version of the strategy method to elicit these decisions.^12^ Rather than eliciting their choices for each table (for senders) and each message (for receivers), participants were just presented one instance and were told that experimenters would infer from their choice that their behavior would have been analogs in the other eventuality. That is, that we would interpret that senders who lied (were honest) when the table selected was A would have also lied (been honest) if the table selected had been B, and viceversa. Similarly, when playing as receivers, subjects were told we would interpret that if they followed (distrusted) a message saying that the table selected was A, they would have equally followed (distrusted) a message reporting that the table selected was B (and viceversa).

In the punishment treatment, receivers were presented four additional choices. They had to decide whether they would accept or reduce the payoffs to zero for each of the four possible histories of the game, i.e., {*m* = θ, *s* = θ}, {*m*≠θ, *s* = θ}, {*m* = θ, *s*≠θ} and {*m*≠θ, *s*≠θ}.

Participants recorded their choices in paper booklets, one booklet per treatment. Each page of the booklet presented a decision round. Subjects were not allowed to move to a new decision round until all participants had finished with that round. In each treatment, we elicited beliefs about the percentage of senders in the session who would send truthful messages and the percentage of receivers who would follow the sender's message: We paid 100 extra points to subjects whose guess was within a 5 percentage points band around the actual target percentage. In the punishment treatment, we also elicited beliefs about the percentage of receivers participants expected to reduce payoffs in each of the four possible histories. The order of decision rounds within each treatment was: (1) choice as receiver; (2) choice as sender; (3) elicitation of beliefs about expected truth and trust rates. In the punishment treatment there were two additional rounds: (4) punishment choices and (5) elicitation of beliefs about punishment rates.

At the end of each session, participants were called one by one to an adjacent room where morphometric measurements were taken in private by one experimenter and two research assistants. After this, one treatment was selected for payment. Roles were randomly assigned within each anonymously matched pair of participants and payoffs were determined according to their decisions. Subjects were paid their earnings in cash in addition to a 5€ show-up fee for this experiment. The exchange rate between points in the experiment and money was 100 points=1€. Average earnings were 7.82€.

## 4. Results

### 4.1. Descriptive Statistics

Table 3 contains descriptive statistics for the three masculine physical traits we consider. They correlate only slightly. The Spearman's correlation coefficient between fMM and fWHR is 0.126 (*p* = 0.099, *n* = 167) and between fMM and 2D:4D is -0.128 (*p* = 0.105, *n* = 167). No significant correlation exists between fWHR and 2D:4D. These weak correlations are in line with previous studies (Sanchez-Pages et al., 2014), and were expected since masculinity is not a latent concept but a set of traits typical of males. fWHR is non-dimorphic so it was not expected to correlate with the other two traits, which are sexually dimorphic. 2D:4D is a measure of prenatal testosterone and fMM of adolescent testosterone. These two periods of exposure to sexual hormones independently influence adult behavior (Berenbaum and Beltz, 2011)^13^.

**Table 3:** Descriptive statistics.

**Variable**	**Mean**	**Std dev**	**Min**	**Max**	**n**
1.2D:4D	0.961	0.027	0.887	1.059	168
2. fWHR	1.929	0.116	1.584	2.260	168
3. fMM	0.093	0.021	0.047	0.156	167
4. BMI	23.458	3.008	15.570	34.478	168
5. Age	21.940	2.299	18	29	168

### 4.2. Aggregate Behavior

The percentages of untruthful messages in the control (37.5%) and punishment (25%) treatments were well below the theoretical prediction of 50% (Proportion test, *p* = 0.001 and *p* < 0.001, respectively, *n* = 168). They were also significantly different from each other (*p* = 0.013, *n* = 336). Trust rates were 63.1% in the control treatment and 58.9% in the punishment one. Both rates were significantly higher than 50% (*p* < 0.001 and *p* = 0.020, respectively, *n* = 168) and similar to those in Sanchez-Pages and Vorsatz (2007), but not different from each other. Beliefs about trust rates were very accurate, 61.4% and 58.1% in the control and punishment treatments, respectively. The distributions of beliefs were not different across treatments (Mann-Whitney, *p* = 0.245, *n* = 335).

Table 4 summarizes the proportion of subjects in each category of our behavioral taxonomy by treatment. Note that frequencies do not add up to 100% vertically because some behavioral classifications overlap. The first result stemming from this table is that deception is more common than lying. Altruistic lies are rare whereas deception by truth-telling is quite frequent and seems unaffected by the threat of punishment. The second result is that the possibility of punishment reduces the frequency of lies; as mentioned earlier, the difference in the percentage of untruthful messages between the two treatments is statistically significant. Selfish lying accounts for just over half of all instances of deception in the control treatment but only accounts for about a third in the punishment one. The third and last result is that a substantial proportion of subjects can be classified as strong truth-tellers. The frequency of this behavior differs across treatments (*p* = 0.033, *n* = 335), suggesting that the possibility of punishment induced senders to switch from selfish lying to costly truth-telling. The threat of punishment was indeed very real: The punishment rate after history {lie,trust} was substantial, 27.38%.^14^

**Table 4:** Frequency of behavioral types by treatment.

**Behavior \ Treatment**	**Control (%)**	**Punishment(%)**
Lying	37.5	25
Deception	55.8	46.5
By lying	29.2	17.6
By truth-telling	26.6	28.9
Strong truth-telling	35.7	47.9
Altruistic lying	8.5	5.6

### 4.3. (Dis)honesty

We next use regression analysis to study the association between lying and deception on the one hand and the three masculine physical traits we consider on the other. In Table 5 below, we present the results of five random-effects regressions with robust standard errors clustered at the session level. These models pool the data from the two treatments and include a dummy variable for the punishment one. The three masculine traits and beliefs about the trust rate among receivers (when used as a control) are standardized. Coefficients should then be interpreted as the change in the outcome variable produced by a one standard deviation change in the corresponding independent variable.

**Table 5:** Random-effects models.

	**Belief**	**Lie**	**Lie**	**Lie**	**Lie**
				**(Trust <50%)**	**(Trust>50%)**
	**(1)**	**(2)**	**(3)**	**(4)**	**(5)**
fWHR	2.276^*^	–0.058^*^	–0.066^*^	–0.091^**^	–0.044
	(0.100)	(0.083)	(0.051)	(0.031)	(0.206)
fMM	3.497^*^	0.039^*^	0.032	0.088	0.038
	(0.079)	(0.064)	(0.107)	(0.228)	(0.254)
2D:4D	–3.169^**^	–0.061^**^	–0.054^*^	0.023	0.095^**^
	(0.027)	(0.023)	(0.051)	(0.296)	(0.049)
Punishment	–3.193	–0.125^***^	-0.112^**^	-0.068	-0.146^*^
	(0.395)	(0.008)	(0.017)	(0.223)	(0.062)
Belief			0.067^***^	–0.018	0.037
			(0.002)	(0.866)	(0.593)
Observations	333	334	333	103	191

*All specifications control for the BMI and age of the subject. Robust standard errors*.

*clustered at the session level. Variables are standardized. p-values in parentheses*.

*** *denotes p <0.01,*

** *p <0.05,*

* *p <0.1*.

**Beliefs**. Column (1) studies the association between masculine physical traits and participants' beliefs about trust rates in their session. All three markers display a significant coefficient, although in varying degrees of significance and directions. An increase in fMM (fWHR) by a standard deviation increases the expected trust rate by 3.5 (2.3) percentage points (pp). However, higher exposure to prenatal androgens as measured by 2D:4D, decreases the expected trust rate by 3.2 pp.

**Result 1:** Higher levels of fMM are associated with senders expecting more receivers to follow their message. Higher exposure to prenatal testosterone and higher fWHR are associated with the opposite.

Note that the coefficients of interest in column (1) are relatively small and on those for fMM and fWHR are weakly significant. This already suggests that the association between beliefs and our masculine traits is not strong. We will come back to this issue below.

**Lying**. In the rest of columns of Table 5, the dependent variable is a dummy with value one if the subject lied. We chose a linear probability model (LPM) for these regressions because we are interested in the marginal effects of the masculine traits. These marginal effects are intuitively measured by the coefficients of a LPM: changes in a variable corresponds to a percentage points (pp) change in the probability of lying. LPMs, however, present two problems: heteroskedasticity in errors (by construction), and estimation bias, which has been shown to increase with the proportion of predicted probabilities outside the [0, 1] interval (Horrace and Oaxaca, 2006). We use clustered robust standard errors to avoid the first issue. On the other hand, only 0.5% of our predicted probabilities is negative and none is above one, suggesting that our LPM estimates are fairly unbiased as well. Nonetheless, we also ran random-effects probit models (see Table A1 in the Appendix), which yielded similar results.

The specification in column (2) estimates the total effect of the three masculinity markers we study on lying. Again, the traits display sizeable effects at different degrees of significance. 2D:4D is associated to a decrease in the probability of lying by 6.1 pp. This regression thus suggests that individuals exposed to more testosterone *in utero* engaged less in lying. The estimates for the other traits are non-negligible but less significant.

Column (3) includes subjects' expected trust rates as a control. The coefficient for fWHR increases in significance and absolute value whereas the one for 2D:4D decreases. An increase of one standard deviation in fWHR now leads to a reduction in lying by 6.6 pp, and an increase in the latter to a decrease by 5.4 pp.

**Result 2**. Higher fWHR and higher exposure to prenatal testosterone are associated to less lying.

The positive coefficient for beliefs implies that participants who believed that a higher fraction of receivers would trust messages were more likely to lie.^15^ The coefficient is highly significant and its size is substantial: a standard deviation increase in the expected trust rate translates into a 6.7 pp increase in the probability of lying.

**Preferences vs. beliefs**. The regression in column (3) is also important because it allows us to explore the extent to which the association between our masculine traits and lying behavior operates through beliefs about the behavior of others, through preferences, or both (Eisenegger et al., 2012). Assuming that sender's behavior depends on preferences, i.e., lying aversion, and beliefs about receivers' behavior and that, in turn, both preferences and beliefs vary with masculine traits implies that column (2) estimate the total association of our biomarkers with dishonesty. When in column (3) we control for participants' beliefs about trust rates we would be estimating the indirect association of the trait via preferences as we would be switching off the beliefs channel. This implies that the differences in estimates between those in columns (2) and (3) allows us to measure the size of the effect of our masculine traits on lying operating through beliefs. The sizes of these effects are all very small, approximately 0.08 pp for a one standard deviation increase in fWHR and –0.07pp for a one standard deviation increase in fMM and 2D:4D. This would corroborate the following result:

**Result 3:** Masculine markers have a statistically significant but weak association with lying via beliefs about the behavior of receivers.

Let us mention that this identification strategy rests on two assumptions. The first one is that beliefs are measured without error. This is important because, as Gillen et al. (2019) have shown, measurement error in a control variable (beliefs in this case) that correlates both with the dependent variable (lying) and other controls (masculine traits) alters estimates. The presence of a substantial measurement error in elicited beliefs would distort the estimates in column (3) and affect our inference of effect sizes. Secondly, we are assuming that the beliefs about trusting rates that individuals report do not depend on their decision as senders. However, it might be the case that the action participants take as senders influence the beliefs they report.

To partially ameliorate these concerns, we report in the Appendix the results of an instrumental variable approach where we substitute elicited beliefs by the residuals from the estimation in column (1) (see Table A2). These residuals are thus the expected trust rates left unexplained by the masculine physical traits we study. The estimates resulting from this exercise are analogs to those in column (3) and can thus be interpreted as the association between the masculine traits and lying via preferences. The coefficients for fMM and 2D:4D become more significant, reinforcing the idea the effect of these traits operate mostly through lying aversion. That said, this IV approach is not a panacea and this result should be taken as suggestive.

**Deception**. At this point, the distinction between lying and deception becomes important. A higher likelihood of sending a truthful message does not necessarily indicates stronger prosociality. If the receiver is expected to distrust the sender, telling the truth becomes a form of sophisticated deception (Sutter, 2009). Columns (4) and (5) account for the different ethical and monetary implications of lying depending on receivers' expected trust rates. These models restrict the analysis to subjects who believed that less (more, respectively) than 50% of receivers would trust their message. We leave out senders who believed that exactly 50% of receivers would follow messages as these senders would be indifferent between lying or not. Due to the reduction in observations, we lose some precision. Still, estimates show that the association between fWHR and lying observed in column (3) is only significant for senders who expected receivers to distrust messages, although coefficients in columns (4) and (5) are not significantly different from each other.^16^ In addition, the coefficient for 2D:4D is only significant for senders whose expected trust rates were above 50%. In addition, it is statistically different from the coefficient in column (4); a *t*-test of the equality of the coefficients returns a *p*-value of 0.008.^17^

**Result 4:** Higher fWHR is associated with more deception by telling the truth, whereas higher exposure to prenatal testosterone is positively associated with strong truth-telling.

To address the possibility of false positives, we also run a series if bivariate regressions where each of the three masculine traits is regressed on the dependent variables. Table A3 in the Appendix shows that almost all coefficients are of the same magnitude and significance as those in Table 5. The only difference is that the association between 2D:4D and beliefs is no longer significant in the corresponding bivariate regression. For the sake of transparency, we also include in the Appendix the analysis of receivers' beliefs, trusting decisions and punishment after history {lie, trust} (Table A4 and A5). There, a significant positive association emerges between fWHR and trusting by receivers, especially when senders were expected to have lied in more than 50% of occasions.

## 5. Discussion

Our results suggest that biology, and masculine physical traits in particular, can help explain the observed individual heterogeneity in honesty in strategic communication. This association seems to operate more strongly through preferences than through beliefs about the behavior of others. Two of the masculine traits markers we study (2D:4D and fWHR) are negatively correlated with lying, but the picture changes when we bring into consideration the expected consequences of messages. This is consistent with recent research on the role of testosterone in social interactions. This hormone makes the seek of social status and dominance a salient motivation, but this goal translates into aggression and competitiveness in some contexts and into prosocial behavior in others (Eisenegger et al., 2011, 2012; Millet, 2011; van Honk et al., 2011b). In our experiment, senders could obtain higher status either by outsmarting the receiver or by following the moral imperative of truth-telling, especially when that was costly.

We next elaborate on how our results relate with other results previously observed in the literature.

### 5.1. The Reduced Empathy Hypothesis

According to the dual-process theory, moral decisions trigger immediate emotional responses such as harm aversion and empathy (see Montoya et al., 2013, and references therein). When striving for status, an awareness of the emotions of others might be detrimental to oneself. In that case, instrumental considerations must override emotional responses in order to clear the path for payoff maximization. Research on behavioral endocrinology suggests that prenatal testosterone exposure is indeed positively associated with decreased empathy, even from a very early age (e.g., Knickmeyer et al., 2005), thus facilitating narrow utility maximization over emotional decision making. Recent evidence also shows that testosterone suppresses the activity of the ventromedial prefrontal cortex (vmPFC), a brain region implicated in moral decision making; it is known that individuals with vmPFC lesions are significantly less likely to experience regret, guilt or embarrassment after violating social norms (Carney and Mason, 2010).

Previous research on the association between masculine facial features and economic behavior is also consistent with this *reduced empathy hypothesis*. Stirrat and Perrett (2010) found that subjects with higher fWHR are more likely to exploit the trust of others in the trust game. Haselhuhn and Wong (2012) and Geniole et al. (2014) found that fWHR is positively related to cheating in non-strategic settings. And Jia et al. (2014) observed a positive relationship between the fWHR of CEOs and their probability of engaging in fraudulent accounting practices.

Our results related to masculine facial features as measured by fWHR are consistent with the reduced empathy hypothesis. Subjects with higher fWHR were more likely to engage in deception by telling the truth (Sutter, 2009), which entails hurting the receiver for profit.

### 5.2. The Status Signaling Hypothesis

Social status is likely to be associated with prosocial behavior when prosociality can signal dominance or higher standing (Eisenegger et al., 2012). For instance, Millet and Dewitte (2009) found that individuals exposed to higher amniotic testosterone concentrations are indeed more generous in the dictator game. Two mechanisms might drive this association. First, it seems that testosterone enhances self-image concerns, leading individuals to make choices which make them feel proud and to avoid those considered dishonorable (Wibral et al., 2012). Second, higher androgens levels suppress the immune system and are feasible only for healthy individuals. In that case, traits and behaviors associated with higher testosterone exposure become signals of superior health and genetic fitness (Puts et al., 2012).

Our results related to prenatal testosterone levels are consistent with this form of status signaling. We find a strong association between 2D:4D and truth-telling. This suggests that androgens exposure during foetal development is related to a pure preference for truth-telling, or alternatively, to a stronger lying aversion (Kartik, 2009; Millet, 2011).

In addition, we find a positive association between prenatal testosterone exposure and costly truth-telling, which is consistent with the idea that honesty in communication under divergent preferences can be seen as a signal of status. Sending a truthful message when most receivers are expected to believe it has a monetary cost compared to lying. Such behavior can in turn be rationalized as a costly signal of higher moral or resource standing (Gintis et al., 2001). This interpretation is also consistent with Weston et al. (2007) and van Honk et al. (2015), who find that testosterone administration reduces selfish misreporting and bluffing, and with the literature suggesting that amniotic testosterone concentrations are positively related with prosociality and altruism (e.g., van den Bergh and Dewitte, 2006; Millet and Dewitte, 2009).

### 5.3. The Dual Role of fWHR

The literature on the fWHR suggests that this trait is linked to antisocial behaviors such as untrustworthiness and cheating. But it remains unclear the extent to which this association is due to individuals adopting these behaviors because of their physical appearance. Wang et al. (2019) suggest that fWHR might have been associated with antisocial behaviors in ancestral environments. The more imposing appearance of men with wider faces may lead them to be less concerned with retaliations to their aggressions (Geniole et al., 2015). That might explain the more aggressive financial policies of CEOs with higher fWHR (Mills and Hogan, 2020). In our experiment, males with wider faces may have felt that their deception was less likely to meet a punishment, as it was the case in their daily life, leading them to engage more in deception.

A body of studies offer a more nuanced view on fWHR. Wong et al. (2011) show that firms in the Fortune 500 whose male CEOs have higher fWHR enjoy higher returns-to-assets ratios. This might be due to these individuals being more exploitative, but also to them being more cooperative. In fact, Stirrat and Perrett (2012) show that men with wider faces contribute more to public goods under inter-group competition. In addition, Lewis et al. (2012) find that US presidents with higher fWHR had a higher drive for achievement but were not more aggressive in their policies. This suggests that the link between fWHR and economic behavior might be contingent on the context: Aggression or cheating might be a bad strategy for presidents but it is perhaps useful and/or socially forgiven in business and finance. Alternatively, it might be that fWHR is associated with prosociality when the own group (e.g., a country, a firm) is competing against another. Inter-group competition was absent in our design; that might have shut down any possible association between this trait and honesty.

## 6. Conclusion

In this article, we have offered an exploratory study of the biological roots of lying and deception in strategic communication. Our results suggest that individual differences in honesty in sender-receiver games can be partially explained by physiological factors. We have also explored whether these association operate more strongly through preferences or through beliefs about the behavior of receivers. Exposure to sexual hormones during foetal development increases truth-telling whereas fWHR, which is related to aggression and dominance in a variety of contexts, predicts more lying and more sophisticated deception. We observed these associations in an environment where the preferences of senders and receivers were completely opposed. Future research should explore whether masculine physical traits or other biological markers may a have different relationship with honesty under other preference configurations and contexts.

In addition, further studies on the relationship between biomarkers and dishonesty should include female participants. A next step could be to explore whether hormone levels may relate differently with lying and deception among females. It would also be relevant to study whether the associations between dishonesty and physiology-related traits are mediated by the gender identity of the sender or the receiver once disclosed to the other party.

Finally, it is important to note that our study cannot tease out the direct biological effect of visible masculine traits from their effect on how individuals are perceived and treated by their peers. Any trait an individual displays mixes biological influences (i.e., genes, which respond to the environment through endocrine and nervous system signalling, whose organization also depends on genes and the environment) and the events the individual experiences (including abiotic factors and their interaction with other living beings). From that point of view, the conjecture that prenatal hormone levels may influence human behavior is especially interesting (Beltz et al., 2011; Berenbaum, 2018). There exist some differences in preferential activities associated with differences in prenatal hormone levels, such as the interest in hitting rather than swinging objects or differences in the attention devoted to objects and people. These differences interact with the social environment to produce behavioral differences in adulthood. In other words, biological and social processes interact with each other and jointly affect development. On the other hand, hormone levels in adolescence influence facial features which in turn may influence how people are perceived and treated, leading to further differences in behavior. In sum, it is extremely difficult to disentangle the direct biological effect of masculine physical traits unless individuals are continuously monitored. This is another open avenue for future research.

## Data Availability Statement

The raw data supporting the conclusions of this article will be made available by the authors, without undue reservation.

## Ethics Statement

The studies involving human participants were reviewed and approved by Autonomous University of Madrid Research Ethics Committee (reference CEI 62-1086). Written informed consent for participation was not required for this study in accordance with the national legislation and the institutional requirements. Written informed consent was obtained from the individual(s) for the publication of any potentially identifiable images or data included in this article.

## Author Contributions

All authors listed have made a substantial, direct and intellectual contribution to the work, and approved it for publication.

## Conflict of Interest

The authors declare that the research was conducted in the absence of any commercial or financial relationships that could be construed as a potential conflict of interest.

## Publisher's Note

All claims expressed in this article are solely those of the authors and do not necessarily represent those of their affiliated organizations, or those of the publisher, the editors and the reviewers. Any product that may be evaluated in this article, or claim that may be made by its manufacturer, is not guaranteed or endorsed by the publisher.

## Footnotes

^1^For a survey of the experimental literature on non-strategic communication, see Abeler and Raymond (2019).

^2^Only 1 in 3 financial advisors in the US, 1 in 4 board members in European companies and 1 in 5 US congresspersons are women (UN WOMEN, 2014; Bureau of Labor European Comission, 2016; Statistics, 2017).

^3^Deception games (e.g., Gneezy, 2005) differ from the family of sender-receiver games our design belongs to in that the receiver does not know the set of payoffs in deception games but does in ours.

^4^Two other criticisms of this measure is that sex differences in 2D:4D might be the result of an allometric shift in shape (Kratochvíl and Flegr, 2009; Lolli et al., 2017) and that 2D:4D changes across life (McIntyre et al., 2005; Trivers et al., 2006). However, very recently, Butovskaya et al. (2021) have observed in a very large sample (>7000) across different ethnicities and ages that 2D:4D is stable during life and that sex differences persist after controlling for allometry issues.

^5^We surveyed many of them in Sanchez-Pages et al. (2014).

^6^Because our sample only comprises men, we use male pronouns throughout the paper.

^7^See Sanchez-Pages and Vorsatz (2007) for a formal proof of these results.

^8^Sutter (2009) called the latter *sophisticated deception*.

^9^Erat and Gneezy (2012) call these lies *altruistic white lies*.

^10^Stirrat and Perrett (2010) detected a Spearman correlation *r*_*s*_ = −0.34 between fWHR and trustworthiness. We needed 106 observations to detect that correlation in the present study with α = 0.05 and β = 0.90. Sanchez-Pages and Turiegano (2013) detected an effect of size *d* = 0.409 of fMM on rejections of a low offer in the Ultimatum Game; assuming that 56% of messages would be truthful, as observed in Sanchez-Pages and Vorsatz (2007), 210 observations were needed to detect the same effect size under identical α and β. Finally, Sanchez-Pages et al. (2014) found a Pearson correlation ρ = −0.164 between fMM and bids in a first-price auction. We needed 314 observations to detect that correlation.

^11^That second experiment was on cooperation and third party punishment. Results are reported in Rodriguez-Ruiz et al. (2019), although subjects in the present study who took part in that experiment constituted a small part of the overall sample. For the purpose of that second experiment, we collected data on upper body strength, fluctuating asymmetry, self-perceived attractiveness and sexual orientation. We do not test or report these measures as they are not masculinity traits nor have a previously described association with dishonesty.

^12^The evidence on the differential effect of the strategy method and the direct response method in sender-receiver games is scant and mixed. López-Pérez and Spiegelman (2013) found no significant differences whereas Minozzi and Woon (2020) observed increased overcommunication under the strategy method. We find that average behavior is similar to that in Sanchez-Pages and Vorsatz (2007), who employed the direct response method. Note, however, that we are interested on whether truth-telling relates to masculinity markers rather than on truth-telling rates themselves.

^13^The evidence on prenatal hormone effects in human and non-human primates shows that androgens are the masculinizing agent whilst oestrogens affect both sexes *in utero*. In contrast, there are dramatic sex differences in both androgen and oestrogen exposure during puberty. It has been proposed that puberal exposure acts by refining the organizational effect of sexual hormones during early development (Montoya et al., 2013).

^14^The rest of punishment rates were 9.52% for {truth,distrust}, 4.77% for {lie, distrust} and 2.97% for {truth,trust}.

^15^This is in line with Gneezy (2005) and Peeters et al. (2015) who found that the higher the expected costs of truth-telling, that is, the more trusting receivers are expected to be, the more likely are senders to lie.

^16^We ran a version of this model were the three masculine traits were interacted with a dummy taking the value 1 if the subject expected more than 50% of receivers to follow messages. This model returned a *p*-value of 0.145 for the t-test of the coefficient on the interaction of the expected trust dummy and the subject's fWHR being zero.

^17^This *p*-value is from the *t*-test of the coefficient on the interaction between the expected trust dummy and the subject's 2D:4D being equal to zero in the interacted version of the model.
