# Supplementary material for: Masculinity and Lying
Source: Front Psychol. 2021 Jul 30;12:684226. doi: 10.3389/fpsyg.2021.684226 (PMC8360853; doi:10.3389/fpsyg.2021.684226)
Supplement: Supplementary file 1 [file Data_Sheet_1.PDF]

INSTRUCTIONS FOR THE FIRST PART

We begin with a brief instruction period in which we will give you a general description of the first part of the experiment.

In this part of the experiment, you must take a number of choices in two scenarios we will present to you. You will have to record your choices in the pages you will find in this booklet. During this part of the experiment you will be anonymously matched with another participant.

In each scenario there will be two phases. In the first phase you will have to answer about how you would act in that scenario. In the second phase, you will have to answer about how you think other participants acted in the first part of the scenario

To determine payoffs, we will choose one of the first phases of the two scenarios presented and one of the second phases. For instance, if phase A1 is selected, then the first part of your payment will depend on the choices you and the participant you were matched with took in that phase. Then, we will randomly select one of the second phases, for instance B2. We will add to your previous payment an amount which will depend on how accurate your answer was compared to the behaviour of participants in phase B1.

All payments will be expressed in points. 1 point is equivalent to 1 euro cent. Therefore, 100 points are equivalent to 1 euro. In addition to these payments, you will earn 5 euros for your participation.

Next you will find a page by page description of the scenarios proposed and a series of questions about how you would choose in each of the possible situations (phase 1) and about how you think other participants answered (phase 2). It is very important that you read all instructions carefully. If you think you did not understand something, please, do not hesitate to ask. If you take your decisions without understanding the scenarios, you will earn less money and the data will be useless to us.

If you have any doubt, raise your hand and an instructor will assist you. Now, turn the page to start the experiment.

SCENARIO A

Please, write here your participant number: _______________

In this scenario, there are two roles, sender and receiver, which will be taken up by you and the person you have been anonymously matched with. We will assign these roles with the toss of a coin. This will not be revealed until the end of the experiment. Hence, you will have to respond about how you would act in both roles. For instance, if the toss of the coin determines that you are the sender, your payment in this phase will depend on your answer in the role of the sender and the answer of your partner as receiver.

Before the session, we tossed a coin to select which of the following two tables is going to determine payoffs in this scenario.

Action A	Action B
40 for the sender<br>100 for the receiver	100 for the sender<br>40 for the receiver

Table A

Action A	Action B
100 for the sender<br>40 for the receiver	40 for the sender<br>100 for the receiver

Table B

Only the sender will be informed about the table selected. The sender will have to send a message to the receiver informing him about the table selected. TABLE A or TABLE B. Notice that the sender can send the message he wants. The interpretation of these messages is “The table selected is A” and “The table selected is B”. The receiver observes the message sent by the sender and must decide which action to take, ACTION A (that is, select the left column) or ACTION B (that is, select the right column). The interpretation of these actions is “I think the table selected is A” and “I think the table selected is B”.

Payments for the sender and the receiver are determined by the numbers in the corresponding cell, and hence depend only on the table selected by the coin toss and by the action of the receiver. For instance, if the receiver takes ACTION A and the table selected is TABLE B, then the sender will receive 100 points and the receiver 40 points.

If you have any question about this scenario, raise your hand.

In the next page, you will start answering about how you would behave in this scenario.

SCENARIO A
FIRST PHASE
QUESTIONS AS RECEIVER

Suppose you have been assigned the role of receiver.

For your convenience, we reproduce the two payment tables.

Action A	Action B
40 for the sender<br>100 for the receiver	100 for the sender<br>40 for the receiver

Table A

Action A	Action B
100 for the sender<br>40 for the receiver	40 for the sender<br>100 for the receiver

Table B

Given that you are the receiver, you do not know which table has been selected. The only information you have is the message of the sender.

Suppose that the message of the sender is that the table selected has been TABLE B.

Which action would you take? (circle your answer).

ACTION A ACTION B

IMPORTANT: In the question above, you are only answering how you would choose if the message of the sender is TABLE B, but not how you would choose if the message is TABLE A. To determine your payoff, we will use your choice to infer what action you would have chosen if you had observed the other message. For instance, if you take ACTION A we will infer that you would have chosen ACTION B if you had received the message TABLE A. Similarly, if you take ACTION B we will infer that you would have chosen ACTION A if you had received the message TABLE A. This same procedure will be employed in all your decisions and in all scenarios.

SCENARIO A
FIRST PHASE
QUESTIONS AS SENDER

Suppose you have been assigned the role of sender.

For your convenience, we reproduce the two payment tables.

Action A	Action B
40 for the sender<br>100 for the receiver	100 for the sender<br>40 for the receiver

Table A

Action A	Action B
100 for the sender<br>40 for the receiver	40 for the sender<br>100 for the receiver

Table B

Suppose that the coin toss before the experiment determined that the table selected is: TABLE B.

Which message you send to the receiver? (circle your answer)

TABLE A TABLE B

IMPORTANT: In the question above, you are only answering how you would choose if the table selected is TABLE B, but not how you would choose if the table selected is TABLE A. To determine your payoff, we will use your choice to infer what message you would have sent if you had observed the other table. In particular, if you send the message TABLE A, we will infer that you would have sent message TABLE B if the table selected had been TABLE A. Similarly, if you send the message TABLE B we will infer that you would have sent message TABLE A if the table selected had been TABLE A. This same procedure will be employed in all your decisions and in all scenarios.

SCENARIO A
SECOND PHASE

In this second phase, we will ask you to guess how other participants behaved in the first phase.

Your payments will depend on how accurate your answers are compared to what happened in the first phase of this scenario. For each answer that is within a 5% range above or below the correct answer you will receive 100 points.

Suppose that the table selected is TABLE B. Out of 100 senders, how many senders do you think have sent message TABLE B? _________

Suppose that the message of the sender is TABLE B. Out of 100 receivers who received that message, how many do you think have taken ACTION B? __________

You have finished the first scenario. Please, raise your hand to notify the instructors.

SCENARIO B

Please, write here your participant number: ______________

This scenario is similar to the previous one. We ask you to carefully read the following instructions anyway.

In this scenario, there are two roles, sender and receiver, which will be taken up by you and the person you have been anonymously matched with. We will assign these roles with the toss of a coin. This will not be revealed until the end of the experiment. Hence, you will have to respond about how you would act in both roles. For instance, if the toss of the coin determines that you are the sender, your payment in this phase will depend on your answer in the role of the sender and the answer of your partner as receiver.

Before the session, we tossed a coin to select which of the following two tables is going to determine payoffs in this scenario.

Action A	Action B
40 for the sender<br>100 for the receiver	100 for the sender<br>40 for the receiver

Table A

Action A	Action B
100 for the sender<br>40 for the receiver	40 for the sender<br>100 for the receiver

Table B

Only the sender will be informed about the table selected. The sender will have to send a message to the receiver informing him about the table selected. TABLE A or TABLE B. Notice that the sender can send the message he wants. The interpretation of these messages is “The table selected is A” and “The table selected is B”. The receiver observes the message sent by the sender and must decide which action to take, ACTION A (that is, select the left column) or ACTION B (that is, select the right column). The interpretation of these actions is “I think the table selected is A” and “I think the table selected is B”.

In addition to his decision regarding which action to take, the receiver has the option to *accept* the resulting distribution of payoffs or to *reduce* both his own payoff and that of the sender to zero.

For instance, if the receiver takes ACTION A and the table selected is TABLE B, then the sender will receive 100 points and the receiver 40 points. But if the receiver chooses to *reduce* then both participants will finally obtain zero points in this phase.

SCENARIO B
FIRST PHASE
QUESTIONS AS RECEIVER

Suppose you have been assigned the role of receiver.

For your convenience, we reproduce the two payment tables.

Action A	Action B
40 for the sender<br>100 for the receiver	100 for the sender<br>40 for the receiver

Table A

Action A	Action B
100 for the sender<br>40 for the receiver	40 for the sender<br>100 for the receiver

Table B

Suppose that the table randomly selected is TABLE A. If the message of the sender is TABLE A and then you have taken ACTION A (which in principle awards 40 points to the sender and 100 points to you), do you want to accept the distribution of payoff or reduce your payoff and the sender's payoff to zero?

ACEPT REDUCE

Suppose that the table randomly selected is TABLE A. If the message of the sender is TABLE A and then you have taken ACTION B (which in principle awards 100 points to the sender and 40 points to you), do you want to accept the distribution of payoff or reduce your payoff and the sender's payoff to zero?

ACEPT REDUCE

Suppose that the table randomly selected is TABLE A. If the message of the sender is TABLE B and then you have taken ACTION B (which in principle awards 100 points to the sender and 40 points to you), do you want to accept the distribution of payoff or reduce your payoff and the sender's payoff to zero?

ACEPT REDUCE

Suppose that the table randomly selected is TABLE A. If the message of the sender is TABLE B and then you have taken ACTION A (which in principle awards 40 points to the sender and 100 points to you), do you want to accept the distribution of payoff or reduce your payoff and the sender's payoff to zero?

ACEPT REDUCE

Finally, suppose that the message of the sender is TABLE A. If this is your only information about the table selected, which action would you take? (circle your answer).

ACTION A ACTION B

SCENARIO B
FIRST PHASE
QUESTIONS AS SENDER

Suppose you have been assigned the role of sender.

For your convenience, we reproduce the two payment tables.

Action A	Action B
40 for the sender<br>100 for the receiver	100 for the sender<br>40 for the receiver

Table A

Action A	Action B
100 for the sender<br>40 for the receiver	40 for the sender<br>100 for the receiver

Table B

Suppose that the coin toss before the experiment determined that the table selected is: TABLE B.

Which message you send to the receiver? (circle your answer)

TABLE A   TABLE B

SCENARIO B
SECOND PHASE

In this second phase, we will ask you to guess how other participants behaved in the first phase.

Your payments will depend on how accurate your answers are compared to what happened in the first phase of this scenario. For each answer that is within a 5% range above or below the correct answer you will receive 100 points.

Suppose the table randomly selected is TABLE A. If the message of the sender is TABLE A and the receiver has taken ACTION A afterwards (which in principle awards 40 points to the sender and 100 points to the receiver), out of 100 receivers in that situation, how many do you think have reduced the payoff of both participants to zero?_________

Suppose the table randomly selected is TABLE A. If the message of the sender is TABLE A and the receiver has taken ACTION B afterwards (which in principle awards 100 points to the sender and 40 points to the receiver), out of 100 receivers in that situation, how many do you think have reduced the payoff of both participants to zero?_________

Suppose the table randomly selected is TABLE A. If the message of the sender is TABLE B and the receiver has taken ACTION B afterwards (which in principle awards 100 points to the sender and 40 points to the receiver), out of 100 receivers in that situation, how many do you think have reduced the payoff of both participants to zero?_________

Suppose the table randomly selected is TABLE A. If the message of the sender is TABLE B and the receiver has taken ACTION A afterwards (which in principle awards 40 points to the sender and 100 points to the receiver), out of 100 receivers in that situation, how many do you think have reduced the payoff of both participants to zero?_________

Suppose that the table selected is TABLE B. Out of 100 senders, how many senders do you think have sent message TABLE B? _________

Suppose that the message of the sender is TABLE B. Out of 100 receivers who received that message, how many do you think have taken ACTION B? __________

You have finished the second scenario. Please, raise your hand to notify the instructors.
