# Supplementary material for: Masculinity and Lying
Source: Front Psychol. 2021 Jul 30;12:684226. doi: 10.3389/fpsyg.2021.684226 (PMC8360853; doi:10.3389/fpsyg.2021.684226)
Supplement: Supplementary file 2 [file Data_Sheet_2.pdf]

# Appendix to
# Masculinity and Lying


Marc Vorsatz
UNED

Santiago Sanchez-Pages
King's College London

Enrique Turiegano
Universidad Autónoma de Madrid

| | Lie | Lie (Follow<50%) | Lie (Follow>50%) |
|---|---|---|---|
| fWHR | -2.235* | -3.886 | -1.594 |
| | (1.241) | (2.638) | (1.433) |
| fMM | 6.295* | 18.792 | 7.974 |
| | (3.614) | (18.585) | (7.069) |
| 2D:4D | 8.717* | -4.720 | 15.281 |
| | (4.828) | (6.568) | (10.237) |
| Punishment | -0.452** | -0.376 | -0.654 |
| | (0.214) | (0.359) | (0.309) |
| Belief | 0.010*** | | |
| | (0.004) | | |
| Belief residuals | | 0.007 | 0.008 |
| | | (0.024) | (0.012) |
| Observations | 333 | 103 | 190 |

All models control for BMI and age. Robust standard errors clustered at the session level in parentheses. *** denotes p<0.01, ** p<0.05, * p<0.1.

Table A1: Random-effects Probit models on the decision to lie.

| | Lie | Lie ($2^{nd}$ stage) | Lie (Follow<50%) | Lie (Follow>50%) |
|---|---|---|---|---|
| | (1) | (2) | (3) | (4) |
| fWHR | -0.066* | -0.060* | -0.093** | -0.041 |
| | (0.051) | (0.084) | (0.022) | (0.226) |
| fMM | 0.032 | 0.042** | 0.086 | 0.043 |
| | (0.107) | (0.037) | (0.206) | (0.206) |
| 2D:4D | -0.054* | -0.063** | 0.026 | -0.100** |
| | (0.051) | (0.020) | (0.379) | (0.028) |
| Punishment | -0.112*** | -0.121*** | -0.066 | -0.151* |
| | (0.017) | (0.010) | (0.242) | (0.054) |
| Belief | 0.067*** | | | |
| | (0.002) | | | |
| Belief residuals | | 0.065*** | 0.017 | 0.035 |
| | | (0.002) | (0.866) | (0.593) |
| Observations | 333 | 333 | 103 | 190 |

All specifications control for the BMI and age of the subject. Robust standard errors clustered at the session level. All variables are standardized. p-values in parentheses. *** denotes $p<0.01$, ** $p<0.05$, * $p<0.1$

Table A2: Random-effects models with instrumented beliefs.

Regression in column (1) is the same as the one in column (3) in Table 2. It is reproduced here for convenience. Column (2)-(4) employ as beliefs the residuals of the regression in column (1) in Table 2.

| | Belief | Lie | Lie (Trust<50%) | Lie (Trust>50%) |
|---|---|---|---|---|
| | (1) | (2) | (3) | (4) |
| fWHR | 2.918*** | -0.045 | -0.079*** | -0.026 |
| | (0.009) | (0.115) | (0.005) | (0.520) |
| | $n = 335$ | $n = 336$ | $n = 103$ | $n = 194$ |
| fMM | 3.718** | 0.030 | 0.075 | 0.011 |
| | (0.046) | (0.259) | (0.146) | (0.754) |
| | $n = 333$ | $n = 334$ | $n = 103$ | $n = 192$ |
| 2D:4D | -2.091 | -0.063** | 0.009 | -0.093** |
| | (0.169) | (0.028) | (0.723) | (0.038) |
| | $n = 335$ | $n = 336$ | $n = 103$ | $n = 194$ |

Robust standard errors clustered at the session level. All variables are standardized. p-values in parentheses. *** denotes p<0.01, ** p<0.05, * p<0.1.

Table A3: Bivariate regressions for each masculine trait.

| | Belief | Trust | Trust | Trust (Truth<50%) | Trust (Truth>50%) |
|---|---|---|---|---|---|
| | (1) | (2) | (3) | (4) | (5) |
| fWHR | 2.415 | 0.059*** | 0.054** | 0.062** | 0.034 |
| | (0.164) | (0.005) | (0.014) | (0.034) | (0.375) |
| fMM | -0.127 | -0.015 | -0.013 | -0.043 | 0.045 |
| | (0.948) | (0.683) | (0.710) | (0.422) | (0.195) |
| 2D:4D | -0.236 | 0.000 | -0.000 | -0.024 | 0.007 |
| | (0.893) | (0.996) | (0.994) | (0.674) | (0.873) |
| Punishment | -0.431 | -0.041 | -0.038 | -0.236** | 0.125 |
| | (0.867) | (0.515) | (0.542) | (0.012) | (0.245) |
| Belief | | | 0.039 | 0.051 | 0.062 |
| | | | (0.085)* | (0.390) | (0.372) |
| Observations | 333 | 334 | 333 | 119 | 171 |

All specifications control for the BMI and age of the subject. Robust standard errors clustered at the session level. All independent variables are standardized. p-values in parentheses. *** denotes p<0.01, ** p<0.05, * p<0.1.

Table A4: Random-effects models of receiver behavior.

The dependent variable in column (1) is the participant's belief about the fraction of messages in their session they believed were truthful. The dependent variable in the other columns is whether the subject trusted the sender's message when playing as receiver.

| | Belief | Punish | Punish |
|---|---|---|---|
| | (1) | (2) | (3) |
| fWHR | -0.076 | 0.013 | 0.019 |
| | (0.743) | (0.538) | (0.376) |
| fMM | 4.495 | 0.063 | 0.027 |
| | (0.169) | (0.294) | (0.059) |
| 2D:4D | 3.358 | 0.017 | -0.009 |
| | (0.370) | (0.767) | (0.787) |
| Belief | | | 0.253*** |
| | | | (0.000) |
| Observations | 167 | 168 | 167 |

All specifications control for the BMI and age of the subject. Robust standard errors clustered at the session level. All variables are standardized. p-values in parentheses. *** denotes p<0.01, ** p<0.05, * p<0.1.

Table A5: OLS models of punishment after history {lie,trust}.

The dependent variable in column (1) is the participant's belief about the fraction of participants in their session who would choose to reduce payoffs as receivers after history {lie,trust}. The dependent variable in the other columns is whether the subject chose to punish as receiver if the history of the game had been {lie,trust}.
